# Receptor–Ligand Biomarker Profile in Ankylosing Spondylitis: Associations Between *AXL*/*MERTK* Expression, Circulating GAS6 and Protein S Levels, and Disease Activity

**DOI:** 10.3390/life16071066

**Published:** 2026-06-26

**Authors:** Sevil Ceyhan Dogan, Cemile Zontul, Mert Atas, Esma Ozmen, Gulcihan Cinar Kaya, Ayca Tas, Ahmet Karadag

**Affiliations:** 1 Department of Physical Medicine and Rehabilitation, Faculty of Medicine, Sivas Cumhuriyet University, 58140 Sivas, Türkiye; sevilceyhan@cumhuriyet.edu.tr (S.C.D.); mertatas.18@gmail.com (M.A.); dr_ahmetkaradag@hotmail.com (A.K.); 2 Department of Chemistry and Chemical Processing Technologies Services, Yıldızeli Vocational School, Sivas Cumhuriyet University, 58140 Sivas, Türkiye; cemilezontul@cumhuriyet.edu.tr; 3 Department of Medical Biochemistry, Faculty of Medicine, Nigde Omer Halisdemir University, 51240 Nigde, Türkiye; ozmenesma@ohu.edu.tr; 4 Department of Medical Services and Techniques, Yıldızeli Vocational School, Sivas Cumhuriyet University, 58140 Sivas, Türkiye; gulcihancinarkaya@cumhuriyet.edu.tr; 5 Department of Medical Biochemistry, Faculty of Medicine, Sivas Cumhuriyet University, 58140 Sivas, Türkiye

**Keywords:** ankylosing spondylitis, TAM signaling pathway, *AXL*, *MERTK*, GAS6, protein S, biomarker

## Abstract

(1) Background: Ankylosing spondylitis (AS) is a chronic inflammatory rheumatic disease associated with immune dysregulation. The TYRO3, *AXL*, and MER (TAM) signaling pathway, comprising *AXL* receptor tyrosine kinase (*AXL*), MER tyrosine kinase (*MERTK*), growth arrest-specific 6 (GAS6), and Protein S, is a key regulator of immune homeostasis. This study investigated these receptor–ligand components in patients with AS. (2) Methods: A total of 45 patients with AS and 44 healthy controls were enrolled. Serum GAS6 and Protein S levels were measured by ELISA, and *AXL* and *MERTK* expression levels were analyzed by RT-qPCR. Clinical and inflammatory parameters were also evaluated. (3) Results: ESR, CRP, and IL-6 levels were significantly higher, whereas Protein S levels were significantly lower in patients with AS than in controls. No significant differences were observed in GAS6, *AXL*, or *MERTK* expression levels, although lower expression trends were detected in patients. Logistic regression analysis identified CRP, IL-6, Protein S, and *AXL* expression as variables independently associated with AS. ROC analysis demonstrated significant discriminatory performance for *AXL* expression. (4) Conclusions: Alterations in the TAM signaling pathway, particularly reduced Protein S levels and altered *AXL* expression, may contribute to immune dysregulation and persistent inflammation in AS.

## 1. Introduction

Ankylosing spondylitis (AS) is a chronic inflammatory rheumatic disorder that primarily affects the axial skeleton, leading to progressive inflammation and structural changes, particularly in the sacroiliac joints and spine [1]. The disease typically develops during early adulthood and may progressively result in functional impairment and reduced physical mobility [2]. The pathogenesis of AS is multifactorial and involves a complex interplay between genetic susceptibility, particularly the HLA-B27 allele, environmental factors, and immune system dysregulation [3,4]. Current evidence suggests that in chronic inflammatory conditions such as AS, disease progression is driven not only by excessive production of inflammatory mediators but also by impaired resolution of inflammation resulting from deficiencies in pro-resolving mechanisms [5].

Among the pro-resolving systems that orchestrate inflammatory resolution at the molecular level and restrain uncontrolled immune activation, the TAM signaling pathway is considered one of the most prominent pathways. The TAM receptor tyrosine kinase family, consisting of TYRO3, *AXL*, and *MERTK*, represents a crucial signaling axis involved in the regulation of immune responses and control of inflammation [6]. These receptors are activated by their principal ligands, Growth Arrest-Specific 6 (GAS6) and Protein S, and play a central role in the efficient, non-inflammatory clearance of apoptotic cells through efferocytosis [7,8]. GAS6 and Protein S function as bridging ligands that activate *AXL* Receptor Tyrosine Kinase (*AXL*) and MER Tyrosine Kinase (*MERTK*) by binding to phosphatidylserine exposed on the surface of apoptotic cells. Through this interaction, proinflammatory cytokine production is suppressed, whereas anti-inflammatory responses are promoted [8]. The TAM receptor system is widely recognized as a fundamental regulatory mechanism involved in maintaining immune homeostasis and controlling inflammatory responses [9]. Dysregulation of the TAM receptor system has been linked to enhanced inflammatory responses and increased disease activity in systemic lupus erythematosus, rheumatoid arthritis, and other autoimmune disorders [10,11]. In particular, reduced expression of *AXL* and *MERTK* has been associated with elevated inflammatory markers, while alterations in GAS6 levels have been linked to disease activity, suggesting that the TAM signaling axis may serve as a promising biomarker [12].

This study aimed to investigate the expression levels of *AXL* and *MERTK* genes and the circulating levels of their ligands, GAS6 and Protein S, in patients with AS. In addition, the relationship between TAM signaling pathway components, disease activity, and inflammatory parameters was evaluated to determine their potential clinical relevance. By jointly assessing receptor expression and ligand levels, this study sought to provide further insight into the role of the TAM signaling axis in AS pathogenesis and identify potential biomarkers and therapeutic targets associated with impaired resolution of inflammation.

## 2. Materials and Methods

### 2.1. Study Population

This study included 45 patients diagnosed with AS who were admitted to the Department of Physical Medicine and Rehabilitation at Sivas Cumhuriyet University Research and Application Hospital. The control group comprised 44 healthy volunteers matched to the patient group in terms of age and sex, with no history of rheumatic or chronic inflammatory diseases. The diagnosis of AS was confirmed by a specialist physician based on clinical assessment and the 2016 Assessment of SpondyloArthritis International Society (ASAS) classification criteria [13]. Demographic and clinical characteristics of the participants were recorded. Disease activity and pain severity were assessed using the Bath Ankylosing Spondylitis Disease Activity Index (BASDAI) [14], the Ankylosing Spondylitis Disease Activity Score (ASDAS-CRP and ASDAS-ESR) [15], and the Visual Analog Scale (VAS) [16]. Individuals with concomitant chronic inflammatory disorders, active infections, a history of malignancy, or recent use of immunosuppressive medications were excluded. Healthy controls had no history of systemic inflammatory, autoimmune or rheumatic diseases. Serum and whole blood samples were collected from both patients and controls using biological materials approved for research use by the ethics committee. This study was conducted in accordance with the principles of the Declaration of Helsinki and the institutional biosafety regulations. Ethical approval was obtained from the Sivas Cumhuriyet University Health Sciences Research Ethics Committee prior to the initiation of the study (approval date: 16 April 2026; decision no.2026-04/60).

### 2.2. Collection of Serum and Blood Samples

Following an overnight fast, peripheral venous blood samples were collected from both patients and healthy control participants. Blood samples intended for enzyme-linked immunosorbent assay (ELISA) analyses were drawn into serum biochemistry tubes, whereas samples designated for gene expression analyses were collected in EDTA-containing tubes. Blood samples obtained in biochemistry tubes were centrifuged at 3000 rpm for 10 min to separate the serum, which was subsequently transferred into sterile Eppendorf tubes. Whole blood samples collected for gene expression analyses were preserved under the appropriate storage conditions. All specimens were stored at −80 °C until laboratory analysis.

### 2.3. GAS6 and Total Protein S Protein Assays

Serum levels of GAS6 (Cat. No.: E3257Hu, BT LAB, Jiaxing, China) and Total Protein S (Cat. No.: E1149Hu, BT LAB, Jiaxing, China) were quantified using commercially available enzyme-linked immunosorbent assay (ELISA) kits. According to the manufacturer’s specifications, the assay detection ranges for GAS6 and Total Protein S were 0.3–90 ng/L and 0.05–15 mg/L, respectively. The reported analytical sensitivities were 0.13 ng/L for GAS6 and 0.027 ng/L for Total Protein S. All ELISA procedures were performed in accordance with the manufacturer’s instructions. The plates were automatically washed using a BioTek ELx50 microplate washer (BioTek Instruments, Winooski, VT, USA), and the optical density was measured at 450 nm using a BioTek ELx800 microplate reader (BioTek Instruments, Winooski, VT, USA). To ensure analytical reliability, the intra-assay and inter-assay coefficients of variation were maintained below 8% and 10%, respectively. In addition, low- and high-concentration quality control standards were included on each assay plate to verify the measurement accuracy.

### 2.4. RNA Isolation and cDNA Synthesis

Total RNA was isolated from peripheral venous blood samples collected from patients with AS and healthy control subjects using the EcoPURE Total RNA Kit (Cat. No. E2075) according to the manufacturer’s instructions. RNA concentration and purity were assessed using a NanoDrop spectrophotometer (Green BioResearch, USA) by measuring the absorbance at 260 and 280 nm. Only RNA samples with an A260/A280 ratio between 1.8 and 2.0 were included in the subsequent analyses. Complementary DNA (cDNA) was synthesized from purified total RNA using the SweScript RT I First Strand cDNA Synthesis Kit (Cat. No. G3330-100, Servicebio, Wuhan, China) according to the manufacturer’s protocol. The resulting cDNA samples were stored at −20 °C until gene expression analysis.

### 2.5. Gene Expression Analysis of AXL ve MERTK

Gene expression analysis was performed using 2 × SYBR Green Master Mix (Cat. No. GK10002, GipBio, Shanghai, China) on a LightCycler^®^ Real-Time PCR System (Roche Diagnostics, Mannheim, Germany). Commercially optimized human primer sets specific for *AXL* (Cat. No. PRT-0127-HU, AXACELL Biosassay, Tokat, Türkiye), *MERTK* (Cat. No. PRT-0202-HU, AXACELL Biosassay, Türkiye), and the housekeeping gene GAPDH (Cat. No. PRT-0001-HU) (AXACELL Bioassay, Türkiye) were used according to the manufacturer’s instructions. Each RT-qPCR reaction was prepared in a final volume of 10 µL, consisting of 5.0 µL 2 × SYBR Green Master Mix, 3.7 µL nuclease-free water, 0.15 µL forward primer, 0.15 µL reverse primer, and 1.0 µL cDNA template. The amplification protocol consisted of an initial denaturation step at 95 °C for 5 min (1 cycle), followed by 40 cycles of 95 °C for 15 s (denaturation), 60 °C for 30 s (annealing), and 72 °C for 10 s (extension). Following amplification, a melt-curve analysis was performed to verify the specificity of the PCR products. Relative gene expression levels were analyzed using the obtained Ct (cycle threshold) values via the RT^2^ Profiler PCR Array Data Analysis software (Version 3.5, QIAGEN). The software calculated the relative expression changes between patient and control groups by applying the 2^−ΔΔCt^ method after performing normalization using a reference gene. The results were presented as fold-change values, representing the relative change compared to the control group. Genes with a fold-change value greater than 1 were considered upregulated, while those with a fold-change value less than 1 were considered downregulated.

### 2.6. Statistical Analysis

All statistical analyses were performed using SPSS software (version 23.0; IBM Corp., Armonk, NY, USA). Graphical representations of the data were prepared using GraphPad Prism (version 8.0.1; GraphPad Software, San Diego, CA, USA). The normality of the continuous variables was assessed using the Shapiro–Wilk test. Continuous variables are expressed as mean ± standard deviation, minimum and maximum values, or median and interquartile range [IQR], according to the distribution characteristics of the data. Categorical variables are presented as numbers and percentages. Comparisons between patients with AS and healthy controls were performed using the independent samples Student’s t-test for normally distributed variables and the Mann–Whitney U test for non-normally distributed variables. Categorical variables, including sex distribution, HLA-B27 status, and treatment characteristics, were analyzed using the chi-square test. Gene expression levels of *AXL* and *MERTK* were evaluated using Ct values and fold-change analysis, and comparisons were performed between the patient and control groups, as well as according to HLA-B27 status, where appropriate. Multivariate binary logistic regression analysis was conducted to determine the independent factors associated with ankylosing spondylitis. The model included inflammatory markers, serum protein levels, gene expression parameters, and demographic variables. Regression coefficients, standard errors, odds ratios [Exp(B)], and 95% confidence intervals were reported for the results. The diagnostic performance of *AXL, MERTK*, GAS6, and Total protein S in distinguishing patients with AS from healthy controls was assessed using receiver operating characteristic (ROC) curve analysis. The area under the curve (AUC), 95% confidence interval, optimal cutoff value, sensitivity, specificity, positive predictive value, and negative predictive value were calculated. The Youden index was used to determine the optimal cutoff values. Statistical significance was set at *p* < 0.05 for all analyses.

## 3. Results

### 3.1. Demographic and Clinical Characteristics

The study included 45 patients diagnosed with AS and 44 healthy controls with similar age and sex characteristics. The demographic, clinical, and biochemical characteristics of the patient and control groups were compared and evaluated. The mean ages of the control and patient groups were 43.13 and 41.53 years, respectively, and no statistically significant difference in age was found between the groups (*p* = 0.173). Similarly, no statistically significant difference was found in terms of sex distribution between the study groups (*p* = 0.444) (Table 1). The control group comprised 27 men (61.4%) and 17 women (38.4%), whereas the patient group comprised 24 men (53.3%) and 21 women (46.7%). In the patient group, HLA-B27 positivity was detected in 26 patients (57.8%) and HLA-B27 negativity in 19 patients (42.2%). A total of 39 patients (86.7%) were receiving anti-TNF therapy, and six patients (13.3%) were using combination NSAID + DMARD therapy (Table 1). The mean Body Mass Index (BMI; kg/m^2^) of the control and patient groups was 26.93 and 29.11, respectively. A statistically significant difference was found between the groups in terms of BMI values, and it was observed that BMI levels were higher in the patient group compared to the control group.

In the patient group, the mean disease duration was 11.47 ± 6.93 years and the mean time to diagnosis was 8.11 ± 4.90 years. The mean VAS score of the patients was 6.64 ± 2.34, BASDAI score was 4.80 ± 1.93, ASDAS-CRP score was 2.78 ± 0.95, and ASDAS-ESR score was 2.65 ± 0.79. Disease duration ranged from 1 to 25 years, and time to diagnosis ranged from 1 to 22 years. The VAS values ranged from 1.00 to 10.00, BASDAI values from 0.00 to 8.90, ASDAS-CRP values from 1.00 to 4.90, and ASDAS-ESR values from 1.00 to 4.00 (Table 2).

### 3.2. Protein and Gene Expression Levels

In the patient group, ESR, CRP, and IL-6 levels were found to be statistically significantly higher than those in the control group (*p* = 0.016, *p* = 0.001, and *p* = 0.001, respectively). Conversely, total protein S levels were significantly lower in the patient group than in the control group (*p* = 0.040). There was no statistically significant difference between the patient and control groups in terms of GAS6 levels (*p* = 0.271) (Table 3). Furthermore, when total protein S and GAS6 protein levels were compared between HLA-B27 negative and HLA-B27 positive patient groups, no statistically significant difference was found between the two groups in terms of these proteins (*p* = 0.704; *p* = 0.602).

**Figure 1:**
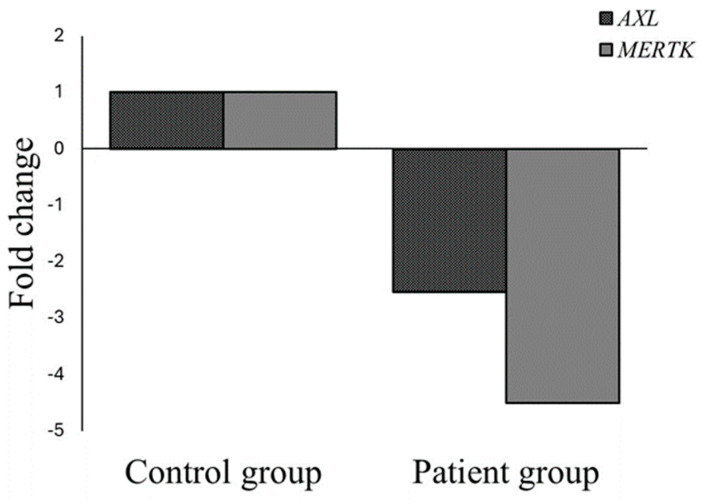
Comparison of Expression Levels of *AXL* and *MERTK* Genes in Patients and the Control Group. *MERTK*, MER tyrosine kinase; *AXL*, *AXL* receptor tyrosine kinase.

No statistically significant difference was found between the patient and control groups in terms of *AXL* and *MERTK* gene expression levels (*p* = 0.689 and *p* = 0.169, respectively). Although not statistically significant, *AXL* and *MERTK* gene expression levels were approximately 2.5-fold lower and 4.5-fold lower, respectively, in the patient group than in the control group (Figure 1).

**Figure 2:**
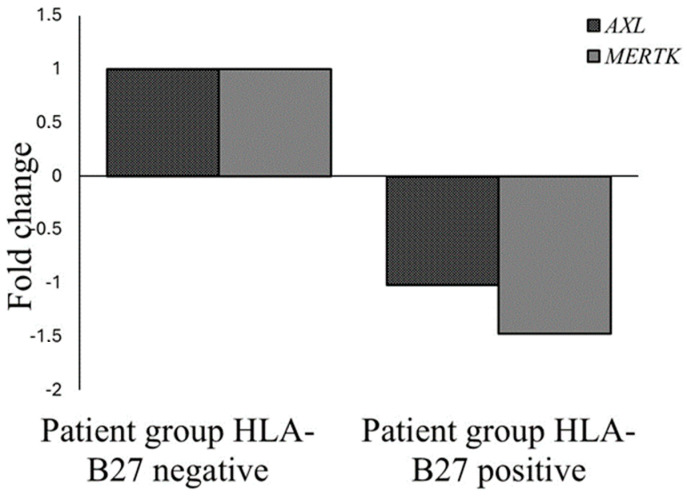
Comparison of *AXL* and *MERTK* Gene Expression Levels in a Patient Group Based on HLA-B27 Positive and HLA-B27 Negative Status. *MERTK*, MER tyrosine kinase; *AXL*, *AXL* receptor tyrosine kinase.

A comparison of *AXL* and *MERTK* gene expression between HLA-B27 negative and HLA-B27 positive patient groups revealed a moderate, although not statistically significant, decrease in *AXL* and *MERTK* gene expression levels in the HLA-B27 positive group. A decrease of approximately 1-fold was observed in *AXL* gene expression and approximately 1.5-fold in *MERTK* gene expression in the HLA-B27 positive group. Furthermore, the decrease in expression was more pronounced for *MERTK* (Figure 2).

To identify independent factors associated with AS, a multivariable binary logistic regression analysis was performed including not only the clinical and biochemical parameters that showed significant differences between the groups, but also the expression levels of *AXL* and *MERTK* genes, which were considered potential contributors to the pathogenesis of the disease. As shown in Table 4, the generated model was statistically significant (Omnibus test, *p* < 0.001). The explanatory power of the model was determined as Nagelkerke R^2^ = 0.597, and the overall classification accuracy of the model was 83.0%. Logistic regression analysis revealed that CRP level (OR = 1.562; 95% CI: 1.065–2.291; *p* = 0.022), IL-6 level (OR = 3.179; 95% CI: 1.413–7.153; *p* = 0.005), total protein S level (OR = 0.771; 95% CI: 0.630–0.942; *p* = 0.011), and *AXL* Ct value (OR = 1.655; 95% CI: 1.065–2.573; *p* = 0.025) were independently associated with ankylosing spondylitis. No statistically significant relationship was found between sedimentation rate and *MERTK* Ct value (*p* > 0.05). Furthermore, given that high *AXL* Ct values indicate low *AXL* gene expression, decreased *AXL* gene expression was independently associated with ankylosing spondylitis.

Receiver operating characteristic (ROC) curve analysis was performed to evaluate the diagnostic performance of *AXL* and *MERTK* gene Ct values, as well as GAS6 and total protein S levels, in distinguishing patients with AS from healthy controls. *AXL* Ct values demonstrated a statistically significant discriminatory performance (AUC = 0.679; 95% CI: 0.560–0.798; *p* = 0.004). The optimal cut-off value for *AXL* Ct was ≥33.62, yielding a sensitivity of 71.1% and a specificity of 50.0%. Since higher Ct values indicate lower gene expression, this finding suggests that reduced *AXL* expression may be associated with AS.

In contrast, neither *MERTK* Ct values (AUC = 0.480; 95% CI: 0.357–0.603; *p* = 0.746) nor GAS6 levels (AUC = 0.414; 95% CI: 0.295–0.534; *p* = 0.163) showed significant discriminatory performance. Total protein S levels, however, demonstrated statistically significant diagnostic performance (AUC = 0.366; 95% CI: 0.250–0.482; *p* = 0.029). The optimal cut-off value for total protein S was ≤6.65, with a sensitivity of 73.3% and a specificity of 34.1%. The complete ROC analysis results are presented in Table 5. No significant discriminatory performance was observed in terms of GAS6 levels (AUC = 0.414; 95% CI: 0.295–0.534; *p* = 0.163), whereas total protein S levels had a statistically significant diagnostic performance (AUC = 0.366; 95% CI: 0.250–0.482; *p* = 0.029). The cut-off value determined for total protein was ≤6.65, with a sensitivity of 73.3% and specificity of 34.1%.

## 4. Discussion

The significantly reduced serum Protein S levels observed in our study suggest that the TAM receptor system may be affected at the ligand level in AS. Protein S is recognized not only as a component of the coagulation system but also as one of the physiological ligands of the TYRO3 and *MERTK* receptors. Protein S and GAS6 initiate TAM receptor activation by binding to phosphatidylserine exposed on the surface of apoptotic cells, thereby playing a critical role in the resolution of inflammation [17]. Previous studies have demonstrated that Protein S and GAS6 exert complementary functions in *MERTK* signaling. Researchers have reported that both ligands support *MERTK* activation under different biological conditions and act cooperatively to ensure optimal receptor function [18]. Therefore, the decreased Protein S levels detected in our study may represent a factor that can reduce TAM system activity, even in the absence of significant alterations in GAS6 levels.

Activation of TAM receptors triggers several anti-inflammatory signaling pathways, including the PI3K/AKT, ERK, and SOCS pathways. These pathways limit the production of pro-inflammatory cytokines by suppressing Toll-like receptor signaling and inhibiting NF-κB activation [9,17]. Therefore, reduced Protein S levels may weaken these protective signaling networks and contribute to the persistence of chronic inflammation. One of the most important functions of the TAM receptor system is the regulation of efferocytosis, which ensures the non-immunogenic clearance of apoptotic cells. *MERTK* signaling, activated by Protein S and GAS6, promotes the efficient phagocytosis of apoptotic cells by macrophages, thereby facilitating the resolution of inflammatory responses. When this mechanism is impaired, apoptotic cell debris accumulates within tissues, leading to an increased release of damage-associated molecular patterns (DAMPs) and the perpetuation of chronic inflammation. Accordingly, the decreased Protein S levels observed in our study may be associated with insufficient resolution of inflammation and sustained immune activation in patients with AS [8,9].

In our study, a trend toward approximately 2.5-fold reduction in *AXL* gene expression was also observed. Previous studies have demonstrated that the GAS6/TAM system plays a critical role in maintaining immune tolerance and that dysfunction of this pathway is associated with chronic inflammatory diseases [19]. Therefore, the observed tendency toward decreased *AXL* expression may represent an additional indicator of impaired immune homeostasis in patients with AS.

Previous studies have demonstrated that *AXL* and *MERTK* expression in the synovium of patients with rheumatoid arthritis plays a crucial role in the resolution of inflammation and is closely associated with IL-6-mediated inflammatory processes [12]. In our study, the observation of a trend toward reduced *AXL* expression alongside significantly elevated IL-6 levels suggests that similar mechanisms may contribute to the pathogenesis of AS. *AXL* is considered one of the most important members of the TAM receptor family, which is involved in the termination of inflammatory responses. Following GAS6-mediated *AXL* activation, SOCS1 and SOCS3 expression is upregulated, resulting in the suppression of Toll-like receptor (TLR) and cytokine receptor signaling pathways. This mechanism is critical for limiting excessive inflammation [9]. Furthermore, *AXL* signaling has been reported to promote macrophage polarization toward the anti-inflammatory M2 phenotype and facilitate the clearance of apoptotic cells through efferocytosis [8].

Therefore, the trend toward decreased *AXL* expression observed in our study may represent not only a biomarker alteration but also a molecular indicator of impaired regulatory mechanisms involved in the resolution of inflammation. Previous studies have reported that TAM receptors regulate macrophage function and play a pivotal role in the resolution of inflammatory responses. Investigators have suggested that reduced *AXL* and *MERTK* activity may contribute to the persistence of proinflammatory immune responses [20]. Taken together, our findings support the hypothesis that dysregulation of the TAM receptor pathway may be involved in the maintenance of chronic inflammation and sustained immune activation in patients with AS. These findings further support the trends of reduced *AXL* and *MERTK* expression observed in our study. Previous research has demonstrated that *AXL* and *MERTK* regulate macrophage polarization and play essential roles in maintaining anti-inflammatory responses. Reduced expression of these receptors impairs the control of inflammatory processes [21]. In this context, the approximately 4.5-fold decrease in *MERTK* expression observed in our study is consistent with this finding.

Experimental arthritis models have shown that deficiency in either *AXL* or *MERTK* exacerbates inflammatory arthritis and enhances inflammatory cell activation, highlighting the protective role of TAM receptors in arthritic inflammation [22]. Accordingly, the reduced expression of *AXL* and *MERTK* observed in our AS cohort may contribute to the persistence of the inflammatory process.

The GAS6/TAM system plays a significant role in regulating the interplay between inflammation and thrombosis [23]. Given the well-established increase in cardiovascular risk among patients with AS, the reduction in Protein S levels identified in our study may be associated not only with inflammatory mechanisms but also with vascular complications. Furthermore, Malikova et al. demonstrated that TAM receptors may undergo proteolytic cleavage in chronic inflammatory diseases, leading to reduced functional receptor availability. This mechanism may represent one possible explanation for the observation that *AXL* emerged as an independent marker in logistic regression analysis despite the absence of a pronounced difference in expression levels between the study groups [24].

Finally, previous studies have identified the TAM receptor system as a promising source of novel biomarkers and therapeutic targets for autoimmune diseases [25]. In the present study, the independent association of *AXL* with ankylosing spondylitis in the logistic regression model, together with its modest discriminatory performance in ROC analysis, suggests that *AXL* may represent a complementary biomarker candidate rather than a standalone diagnostic marker. When interpreted alongside the reduced Protein S levels, these findings support the hypothesis that dysregulation of the TAM signaling pathway may contribute to AS pathogenesis. However, additional validation in larger prospective cohorts is required before the clinical utility of *AXL* as a biomarker can be established.

## 5. Conclusions

This study provides evidence that alterations in the TAM receptor signaling pathway may be involved in the pathophysiology of ankylosing spondylitis. Patients with AS exhibited significantly reduced serum Protein S levels together with increased inflammatory markers, suggesting impairment of pro-resolving mechanisms that normally contribute to immune homeostasis and the termination of inflammation. Although *AXL* and *MERTK* gene expression differences did not reach statistical significance, both receptors demonstrated a downward trend, and reduced *AXL* expression remained independently associated with AS in the multivariable logistic regression analysis. Furthermore, *AXL* demonstrated modest discriminatory performance in ROC analysis. The combined assessment of receptor expression and ligand levels suggests that dysregulation of the GAS6/Protein S TAM signaling axis may contribute to persistent immune activation and inadequate resolution of inflammation in AS.

Although *AXL* expression did not differ significantly between groups, its independent association with AS in logistic regression analysis and its modest discriminatory performance in ROC analysis suggest that *AXL* may provide complementary molecular information regarding disease-related alterations. However, these findings should be considered exploratory and require validation in larger prospective studies before their clinical relevance can be established. In addition, the relatively small sample size and cross-sectional design of the present study warrant cautious interpretation of the results. Future studies involving larger cohorts and mechanistic investigations are needed to clarify the precise role of TAM signaling in AS pathogenesis and to further evaluate its biological and clinical significance.

## 6. Limitations

Several limitations of this study should be considered. First, the relatively small sample size may have limited the statistical power to detect significant differences in *AXL* and *MERTK* gene expression levels between patients with ankylosing spondylitis (AS) and healthy controls. Second, the cross-sectional design precludes the establishment of causal relationships between alterations in the TAM signaling pathway and disease pathogenesis. Third, gene expression analyses were performed using peripheral blood samples, which may not fully reflect the molecular events occurring within inflamed axial joints and affected tissues. Furthermore, only the mRNA expression levels of *AXL* and *MERTK* were evaluated, whereas receptor protein expression, receptor activation status, downstream signaling molecules, and functional assessments of efferocytosis and TAM receptor activity were beyond the scope of the present study.

Another important limitation is that the majority of patients (86.7%) were receiving anti-TNF therapy at the time of sample collection. Since TNF inhibitors are known to modulate inflammatory signaling pathways, cytokine production, macrophage polarization, and immune-regulatory mechanisms, treatment-related effects on inflammatory biomarkers as well as components of the TAM signaling pathway cannot be completely excluded. To address this potential confounding factor, subgroup analyses were performed according to treatment modality (anti-TNF therapy versus NSAID plus DMARD therapy). No statistically significant differences were observed in serum Protein S, GAS6, *AXL*, or *MERTK* expression levels between the treatment groups. However, because only a small number of patients received non-biologic therapy, the statistical power of this comparison was limited. Therefore, the absence of significant differences should be interpreted with caution and does not completely exclude the possibility that ongoing anti-TNF treatment influenced the observed molecular profiles.

Furthermore, soluble forms of TAM receptors, receptor shedding mechanisms, and additional regulatory mediators involved in the GAS6/Protein S TAM signaling pathway were not evaluated. Finally, the absence of longitudinal follow-up data prevented the assessment of temporal changes in TAM signaling in relation to disease progression and treatment response. Future prospective studies including larger cohorts and treatment-naïve patients are warranted to distinguish disease-related alterations from treatment-induced effects and to further clarify the role of the TAM signaling pathway in AS. Despite these limitations, this study provides novel evidence regarding the receptor–ligand profile of the TAM signaling pathway in AS and establishes a foundation for future mechanistic and large-scale clinical investigations.

## Figures and Tables

**Table 1:** Categorical demographic and clinical characteristics of the patient and control groups included in the study.

Parameter	Group	*n* (%)	*p* Value
Sex (Male/Female)	Patients	24 (53.3%)/21 (46.7%)	0.444 ^a^
	Control	27 (61.4%)/17 (38.6%)	
HLA-B27 positive	Patients	26 (57.8%)	
HLA-B27 negative	Patients	19 (42.2%)	
Anti-TNF treatment	Patients	39 (86.7%)	
NSAID + DMARD treatment	Patients	6 (13.3%)	

^a^ Chi-square test (*n* (%)). HLA-B27 percentages were calculated among patients for whom treatment data were available (*n* = 45). Treatment percentages were calculated among patients for whom treatment data were available (*n* = 45).

**Table 2:** Demographic and clinical characteristics of the patient group.

Parameter	Group	Mean ± SD	Min	Max
Disease duration (years)	Patients	11.47 ± 6.93	1.00	25.00
Diagnosis duration (years)	Patients	8.11 ± 4.90	1.00	22.00
VAS	Patients	6.64 ± 2.34	1.00	10.00
BASDAI	Patients	4.80 ± 1.93	0.00	8.90
ASDAS-CRP	Patients	2.78 ± 0.95	1.00	4.90
ASDAS-ESR	Patients	2.65 ± 0.79	1.00	4.00

VAS, Visual Analog Scale; BASDAI, Bath Ankylosing Spondylitis Disease Activity Index; ASDAS-CRP, Ankylosing Spondylitis Disease Activity Score using C-reactive Protein; ASDAS-ESR, Ankylosing Spondylitis Disease Activity Score using Erythrocyte Sedimentation Rate; SD, Standard Deviation.

**Table 3:** Comparison of ESR, CRP, IL-6, total protein S, and GAS6 levels in patient and control groups.

	Patient Group (*n* = 45) Median [IQR]	Control Group (*n* = 44) Median [IQR]	*p*-Value
ESR (mm/h)	10.00 [10.00]	4.00 [7.50]	0.016 *
CRP (mg/L)	3.08 [7.28]	0.87 [1.03]	0.001 *
IL-6 (pg/mL)	2.76 [2.38]	1.50 [0.85]	0.001 *
Total protein S (mg/mL)	13.71 [7.40]	15.92 [12.68]	0.040 *
GAS6 (ng/mL)	0.89 [1.25]	1.12 [1.84]	0.271

* Statistically significant compared with the control group (*p* < 0.05). ESR, Erythrocyte Sedimentation Rate; CRP, C-reactive protein; IL-6, Interleukin-6; GAS6, Growth Arrest-Specific 6; *MERTK*, MER tyrosine kinase; *AXL*, *AXL* receptor tyrosine kinase; GAS6, Growth Arrest-Specific 6; IQR, Interquartile Range.

**Table 4:** Multivariable binary logistic regression analysis of clinical and molecular variables associated with AS.

Variable	B	S.E.	*p* Value	OR [Exp(B)]	95% CI for OR
CRP	0.446	0.195	0.022 *	1.562	1.065–2.291
ESR	−0.055	0.080	0.490	0.946	0.809–1.107
IL-6	1.157	0.414	0.005 *	3.179	1.413–7.153
Total protein S	−0.260	0.103	0.011 *	0.771	0.630–0.942
*AXL*	0.504	0.225	0.025 *	1.655	1.065–2.573
*MERTK*	−0.005	0.148	0.975	0.995	0.744–1.331

Data are presented as regression coefficient (B), standard error (S.H.), odds ratio [OR = Exp(B)], and 95% confidence interval (CI). OR: Odds ratio; CI: Confidence interval; CRP: C-reactive protein; ESR: Erythrocyte sedimentation rate; IL-6: Interleukin-6; *MERTK*, MER tyrosine kinase; *AXL*, *AXL* receptor tyrosine kinase * Statistically significant at *p* < 0.05.

**Table 5:** ROC Analysis Results of *AXL* and *MERTK* Ct Values, GAS6, and Total Protein S Levels in Differentiating Patients with AS from Healthy Controls.

	*AXL*	*MERTK*	GAS6	Total Protein S
**AUC (%95 GA)**	0.679 (0.560–0.798)	0.480 (0.357–0.603)	0.414 (0.295–0.534)	0.366 (0.250–0.482)
**p değeri**	0.004 *	0.746	0.163	0.029 *
**Cut-off point**	≥33.62	≥33.39	≤0.22	≤6.65
**Sensitivite (%)**	71.1	53.3	68.9	73.3
**Specificity (%)**	50.0	50.0	45.5	34.1
**PPV (%)**	59.3	52.2	56.4	53.2
**NPV (%)**	62.9	51.2	58.8	55.6

AUC, Area under the receiver operating characteristic curve; GA, Confidence interval; PPV, Positive predictive value; NPV, Negative predictive value; Ct, Cycle threshold; GAS6, Growth Arrest-Specific 6; *MERTK*, MER tyrosine kinase; *AXL*, *AXL* receptor tyrosine kinase * *p* < 0.05 was considered statistically significant.

## Data Availability

The original contributions presented in this study are included in the article. Further inquiries can be directed to the corresponding author.

## Abbreviations

The following abbreviations are used in this manuscript:

**Abbreviation**	**Definition**
AS	Ankylosing Spondylitis
*AXL*	*AXL* Receptor Tyrosine Kinase
*MERTK*	MER Tyrosine Kinase
TAM	TYRO3, *AXL*, and *MERTK* Receptor Family
GAS6	Growth Arrest-Specific 6
IL-6	Interleukin-6
CRP	C-Reactive Protein
ESR	Erythrocyte Sedimentation Rate
HLA-B27	Human Leukocyte Antigen B27
BASDAI	Bath Ankylosing Spondylitis Disease Activity Index
ASDAS	Ankylosing Spondylitis Disease Activity Score
ASDAS-CRP	Ankylosing Spondylitis Disease Activity Score Using C-Reactive Protein
ASDAS-ESR	Ankylosing Spondylitis Disease Activity Score Using Erythrocyte Sedimentation Rate
VAS	Visual Analog Scale
ELISA	Enzyme-Linked Immunosorbent Assay
RT-qPCR	Real-Time Quantitative Polymerase Chain Reaction
cDNA	Complementary DNA
RNA	Ribonucleic Acid
Ct	Cycle Threshold
ROC	Receiver Operating Characteristic
AUC	Area Under the Curve
OR	Odds Ratio
CI	Confidence Interval
PPV	Positive Predictive Value
NPV	Negative Predictive Value
BMI	Body Mass Index
PI3K	Phosphoinositide 3-Kinase
AKT	Protein Kinase B
ERK	Extracellular Signal-Regulated Kinase
SOCS	Suppressor of Cytokine Signaling
TLR	Toll-Like Receptor
NF-κB	Nuclear Factor Kappa B
DAMPs	Damage-Associated Molecular Patterns
TNF	Tumor Necrosis Factor
NSAID	Non-Steroidal Anti-Inflammatory Drug
DMARD	Disease-Modifying Antirheumatic Drug
IQR	Interquartile Range
SD	Standard Deviation
